# Mathematical Models for Possible Roles of Oxytocin and Oxytocin Receptors in Autism

**DOI:** 10.1155/2019/7308197

**Published:** 2019-11-11

**Authors:** Mark M. Gottlieb

**Affiliations:** 1700 Shattuck Avenue, #114, Berkeley, CA 94709-3402, USA

## Abstract

This paper develops mathematical models examining possible roles of oxytocin and oxytocin receptors in the development of autism. This is done by demonstrating that mathematical operations on normalized data from the Stanford study, which establishes a correspondence between severity of autism in children and their oxytocin blood levels, generate a graph that is the same as the graph of mathematical operations on a normalized theoretical model for the severity of autism. This procedure establishes the validity of the theoretical model and the significance of oxytocin receptors in autism. A steady-state model follows, explaining the constant baseline concentrations of oxytocin observed in the cerebral spinal fluid and blood in terms of the neuromodulation by oxytocin of oxytocin receptors on the magnocellular neurons that produce oxytocin in nuclei in the hypothalamus. The implications of these models for possible roles of oxytocin and oxytocin receptors in autism are considered for several unrelated conditions that may be associated with autism. These are oxytocin receptor desensitization and downregulation as factors during labor in offspring autism development; reductions in the oxytocin receptor numbers in the fixed oxytocin receptor expression that occurs before birth; MAST Immune System disease; and the excess number of dendritic spines from lack of pruning observed in brains of autistic people. Research into the feasibility of generating magnocellular neurons and other neurons from adult stem cells is suggested as a way of doing *in vitro* studies of oxytocin and oxytocin receptors to assess the validity of theories presented in this paper.

## 1. Introduction

This paper develops mathematical models that demonstrate the significance of oxytocin and oxytocin receptors in the development of autism. The paper establishes the significance of the oxytocin receptor in autism based on mathematical operations on findings by Stanford University [1], in which it is shown that the severity of autism measured on the NEPSYS theory of mind scale is correlated with oxytocin concentrations in the blood of children. The mathematical operations demonstrate that, for fixed values of the oxytocin concentration, the severity of the autism in the Stanford study, as measured on the NEPSYS theory of mind scale, is directly proportional to the fraction of unavailable oxytocin receptors in the brains of the children.

Data from the Stanford study [1] on the severity of autism and oxytocin blood levels in children, which have undergone normalizing mathematical operations, are compared with a normalized theoretical model based on very plausible considerations of autism severity and oxytocin receptor numbers. The theoretical model is based on the observation that the severity of autism can be expressed as being inversely proportional to the product of the relative number of oxytocin receptors and the concentration of oxytocin. In other words, the severity S is assumed to be proportional to 1/(RaO), where Ra is the relative number or abundance of available oxytocin receptors and O is the concentration of oxytocin, i.e., the smaller the relative number or abundance of oxytocin receptors or the smaller the concentration of oxytocin or both, the greater the severity of the autism.

Following the mathematical development indicating the significance of the oxytocin receptor, a steady-state model is introduced as a basis to explain the constant baseline levels of oxytocin in the cerebral spinal fluid and the blood. This model proposes that neuromodulation of oxytocin-producing magnocellular neurons from oxytocin in the cerebral spinal fluid produces oxytocin at a rate in which the rate of oxytocin production is equaled by the rate of oxytocin loss, resulting in a constant baseline oxytocin concentration. The Stanford study mentioned above found that the severity of autism was directly proportional to the baseline oxytocin concentration in the blood of the children. Consequently, knowledge of the baseline concentration of oxytocin and the factors that affect it is important to the understanding of autism.

The remainder of the paper examines four potential causes of autism in terms of possible roles played by oxytocin and oxytocin receptors as factors in the development of autism for each cause. These causes include the following:

- Autism arising from desensitization and downregulation of oxytocin receptors following continuous Pitocin infusion during labor
- Autism arising from the reduction of oxytocin receptors from the fixed expression of oxytocin receptors that occurs before birth
- Autism arising from MAST Immune System disease
- Autism arising from the lack of pruning of dendritic spines

Research into the generation of oxytocin-producing magnocellular neurons from adult stem cells as a means of studying oxytocin receptors is suggested as a method by which the theories presented in this paper can be explored. The paper concludes that oxytocin and oxytocin receptors may be responsible for the diminished social functioning observed in people with autism, while other characteristics of autism may have other causes.

The agreement between the Stanford findings following the mathematical operations on them indicated in the paper and the theoretical model proposed therein strongly argue for the significance of the oxytocin receptor in autism. While much of the science indicated in the paper for the roles of oxytocin and oxytocin receptors in the steady-state model and the four causes of autism mentioned above has not been established, it is contended that these roles for oxytocin and oxytocin receptors are plausible explanations appropriate for this model and these causes, in light of what is known about oxytocin and what has been established about oxytocin receptors in the paper. As such, these roles for oxytocin and oxytocin receptors warrant further consideration to evaluate their suitability, and if validated, research should be undertaken to gain further knowledge about them and to develop treatment protocols addressing them for the causes of autism they impact.

## 2. Empirical and Theoretical Mathematical Models

**Figure 1:**
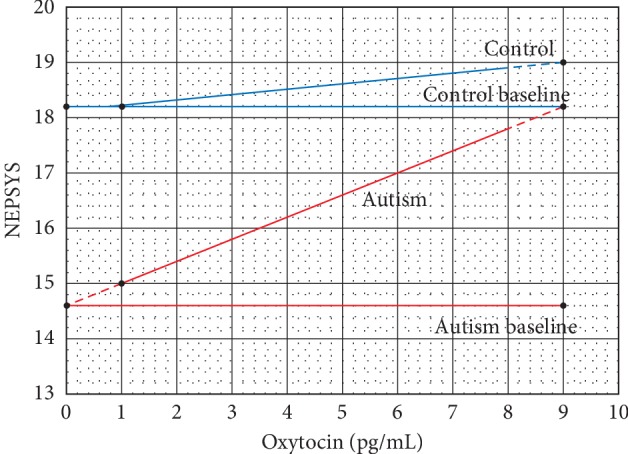
Severity of autism as measured by the NEPSYS theory of mind scores in the Stanford study corresponding to oxytocin concentrations, with indicated baseline levels for controls and the lower limit of autism.

**Figure 2:**
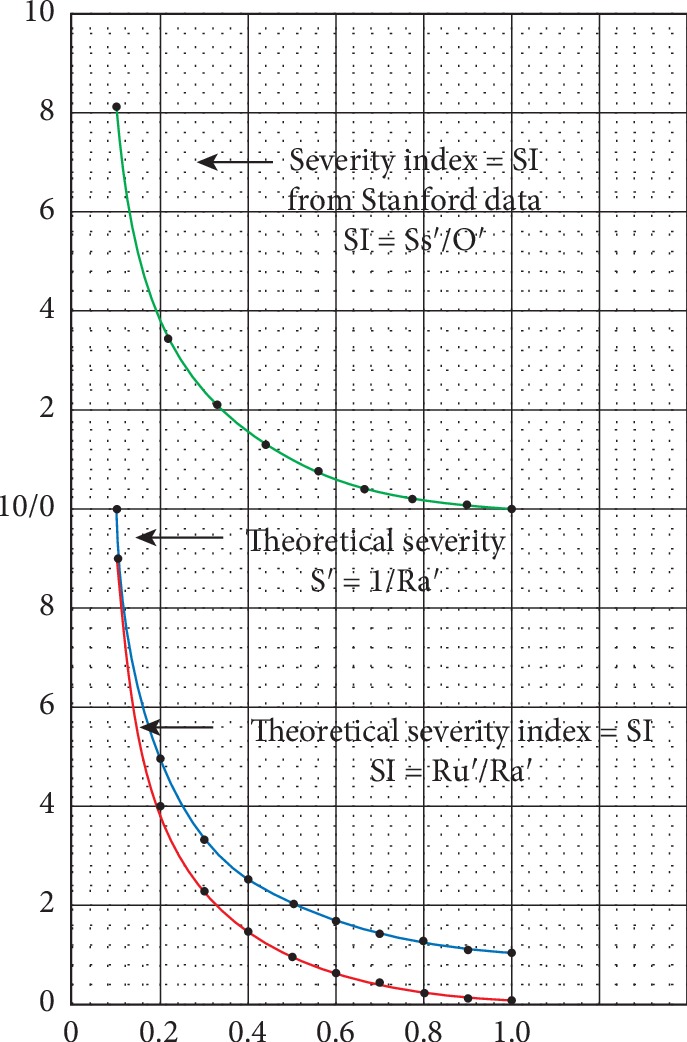
Graphical data showing equivalence of the severity index (SI) for the Stanford data (green) and the adjusted theoretical severity index (SI) (red). Note that, for all values on the *X*-axis, SI = S′ − 1, where the theoretical severity (S′) is calculated as indicated (blue).

Data used in the Stanford study, which established a correspondence between scores on the NEPSYS theory of mind scale and baseline oxytocin concentrations in autistic children, are reproduced in Figure 1 from Figure 2 in reference [1], which is used with the permission of the National Academy of Sciences. Figure 1 reproduces the control and autism lines appearing in Figure 2 in the Stanford study. The autism line is the least squares calculation for the data. The control baseline is established from the assertion in the Stanford paper that an oxytocin concentration of 1 pg/ml on the control line indicates a baseline for normal functioning. The dashed lines for the autism line are extrapolations for the autism line at oxytocin baseline concentrations of 0 pg/ml and 9 pg/ml, where it meets the control baseline. This was determined by printing the Stanford graphs and extrapolating the data for the least squares line to the control baseline. The autism baseline is extended to incorporate the value of the severity of autism, as indicated by the NEPSYS theory of mind scale at a value of 0 pg/ml of oxytocin, the worst-case scenario, and represents the lower limit of the severity of autism on the NEPSYS theory of mind scale.

Normalization is a process in which data are converted to a unitless scale between 0% and 100% or in terms of decimal fractions between 0 and 1 [2]. The data for the severity of autism in terms of the NEPSYS theory of mind score are normalized on a vertical scale from 0 to 1, which indicates the relative severity of the autism Ss′ as measured from the autism line. Ss′, the normalized severity of the autism, is obtained from the least squares calculation for the Stanford data. The normalized value of the severity Ss′ is given by the equation (Cn-An)/(Cn-ABn), where Cn is the value of the control baseline on the NEPSYS theory of mind scale, An is the NEPSYS value for the autism on the autism line from the least squares calculation, and ABn is the value of the autism baseline on the NEPSYS scale. This equation reduces to (Cn-An)/3.6 to obtain the normalized values for Ss′.

The oxytocin concentration (O) is also normalized on a scale of 0 to 1 on the horizontal scale of Figure 1 in a similar manner over the range of oxytocin concentrations from 0 pg/ml to 9 pg/ml, the value where the autism line meets the control baseline. The normalized value of the oxytocin is indicated by O′, i.e., O′ = Om/(9 pg/ml), where Om is the measured value of the oxytocin concentration in the range indicated above. Then, each point on the vertical normalized NEPESYS theory of mind score is divided by the corresponding point on the horizontal normalized oxytocin scale, i.e., Ss′/O′. Doing this over the entire range of values of Ss′ and O′, corresponding to the two normalized scales, gives the upper graph in green in Figure 2. The green graph can be referred to as a severity index SI derived from the data from the Stanford study and represents an empirically derived mathematical model for severity of autism from the Stanford data.

To illustrate this calculation, consider the oxytocin concentration of 1.0 pg/ml in Figure 1 with a NEPSPS value of 3.2 N, where N is a NEPSYS unit, 18.2 N–15.0 N = 3.2 N. The normalized NEPSYS value is 3.2 N/3.6 N = 0.889, where 3.6 N is the range of NEPSYS values from 18.2 N to 14.6 N. Note that the NEPSYS units N drop out of the calculation for the normalized value. 0.889 is the value of Ss′ for this calculation. Now, consider the normalized value of the oxytocin concentration of (1.0 pg/ml)/(9.0 pg/ml) = 0.111, where 9.0 pg/ml is the range of oxytocin concentrations. Note that the unit pg/ml for the oxytocin concentration drops out of the calculation for the normalized value. The value of O′ for this calculation is 0.111. Now, dividing Ss′/O′ = 0.889/0.111 = 8.009 = 8.0 rounded off, which is the value of SI, the severity index established for this calculation. Looking at the green graph in Figure 2, the value of SI = 8.0 corresponds to the value of O′ = 1.0. Calculating the values of SI over the range of O′ from 0.0 to 9.0 yields the green graph in Figure 2.

Now, consider the theoretical model of autism mentioned in this section above as a function of the oxytocin/oxytocin receptor system, in which the severity of the ensuing autism is assumed to be inversely proportional to the product of the normalized concentration of oxytocin O′ and the normalized abundance of the available oxytocin receptors (Ra′), i.e., S′ = 1/(Ra′O′). The abundance of Ra′ and O′ each varies on normalized scales from 0 to 1. So, as the number of oxytocin receptors decreases, the severity would increase, and as the oxytocin concentration decreases, the severity would increase. Now, consider the variation in the severity for constant values of O′, the normalized concentration of oxytocin. This measure of the severity S′ would go as 1/Ra′. This relationship is depicted in the graph in blue in Figure 2. Since this graph arises from a theoretical construct, the coordinate system for this graph can be adjusted by having the horizontal axis moved from 1 to 0, where O′ = 1, while still preserving the functional relationship. So, for any value of O′, S′ − 1 = (1/Ra′) − 1, which is equal to (1 − Ra′)/(Ra′). However, 1 − Ra′ = Ru′, where Ru′ equals the percent or normalized abundance of the unavailable oxytocin receptors. So, S′ − 1 = Ru′/Ra′, yielding the graph in red in Figure 2.

**Figure 3:**
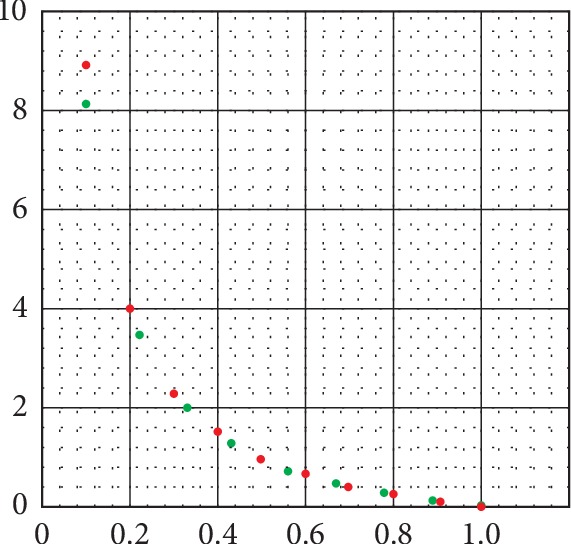
Equivalence of severity indexes (SIs) for theoretical model (red) and for data from the Stanford study (green). Data points for each severity index plot on the same graph indicating the same functional relationship, i.e., Ru′/Ra′ = Ss′/O′ = SI.

To illustrate this calculation, consider a normalized value of Ra = 0.111. Then, Ru = 1.0 − Ra = 0.889. Ru/Ra = 0.889/0.111 = 8.009 = 8.0 rounded off. This is the value of the severity index (SI) on the red graph in Figure 2. Note that this is the same value for the severity index (SI) on the green graph in Figure 2 for this calculation, in which O′ = Ra′ = 0.111 and Ss′ = Ru′ = 0.888, which establishes the equivalence between O′ = Ra′ and Ss′ = Ru′.  Figure 3 illustrates the equivalence of the severity indexes in the red and green graphs in Figure 2 by plotting data from each of them in the same graph. The data for the plots are indicated in Tables 1 and 2.  Figure 3 shows the equivalence of the severity indexes (SIs), by showing that the data for each can be plotted on the same graph, indicating that they have the same functional representation.

In Table 1, Ra′ and Ru′ are normalized values from the theoretical model where Ra′ indicates the normalized value for the available oxytocin receptors and Ru′ indicates the normalized value for the unavailable oxytocin receptors. Ra′ = 1 − Ru′. In Figure 2, Ru′/Ra′ is plotted as a function of Ra′ and is represented by the graph in red termed the severity index (SI), and the data points are indicated in red in Figure 3.

In Table 2, O′ is the normalized value of the oxytocin level O, which varies from 1 to 9 pg/ml. Ss is the number of NEPSYS units between the value for the oxytocin levels on the least squares Autism line in Figure 1 and the control baseline in Figure 1 are taken from the Stanford study. It is a measure of the severity in NEPSYS units associated with this level of oxytocin. Ss′ = Ss/3.6 is the normalized value of the severity Ss in association with the oxytocin levels. SI=Ss′/O′ is the value of the severity index obtained by plotting the normalized value of the severity Ss′ against the normalized oxytocin level O′ and is represented by the graph in green termed the severity index in Figure 2, and the data points are plotted in green in Figure 3. Note that the red and green data points in Figure 3 are on the same graph, showing the equivalence of the calculations they represent.

Also, note that the graphs in green and red in Figure 2 are identical; hence, the severity indexes (SIs) for Ru′/Ra′ as well as for Ss′/O′ are equivalent and will be referred to as the severity index (SI). The graph in green in Figure 2 is derived from a normalization of the data arising from the least square fit of the Stanford measurements of oxytocin levels corresponding to NEPSYS theory of mind score. In other words, it is the graph of Ss′/O′, where Ss′ is the normalized value of the severity of the autism obtained from the least squares calculation of the NEPSYS theory of mind score corresponding to each oxytocin concentration in the range given in Table 2 above of 0 pg/ml to 9 pg/ml, and O′ is the normalized value of the oxytocin concentration. The graph in red in Figure 2 is derived from a normalized and adjusted, or zeroed, theoretical model that is distinct from the Stanford research. The fact that the green and red graphs in Figure 2 are identical indicates the relationship Ss′/O′ = Ru′/Ra′ is valid. Specifically, the normalized severity of the NEPSYS theory of mind score Ss′, which is taken from the least squares calculation of the Stanford data, varies as the normalized number of unavailable oxytocin receptors Ru′ in the theoretical model. Likewise, the normalized oxytocin concentration O′ varies as the normalized number of available oxytocin receptors Ra′ in the theoretical model.

The relationship between Ss′ and Ru′, namely Ss′ = Ru′, alludes to the importance of the oxytocin receptor system in the development of autism. By this model, variation in the number of unavailable oxytocin receptors will have a direct relationship to the severity of autism. The relationship between O′ and Ra′, O′ = Ra′, can be understood in terms the graph of SI, a monotonically decreasing function in the range 0 to 1. As evident from the red and green graphs in Figure 2, for each value of SI, there are unique values of Ss′ and O′ and of Ru′ and Ra′, where Ss′ equals Ru′ and O′ equals Ra′. The relationship of Ss′ and Ru′, indicating the importance of the oxytocin receptor in autism, has been discussed above. The relationship of O′ and Ra′ can be explained as follows: Reducing the oxytocin concentration by a given percentage when the number of oxytocin receptors is fixed which results in the same increase in severity, as reducing the number of oxytocin receptors by the same percentage when the oxytocin concentration is fixed. The normalized values of Ra′ and O′ for each value of the severity index (SI) are equal, i.e., Ra′ = O′, and Ra′ varies as O′ for all the values of O′.

It is important to note the distinction between the severity of autism from the theoretical model, which varies as 1/Ra′O′ and the normalized severity Ss′. Ss′ is taken from the least squares calculation of the variation in the normalized severity of the NEPSYS theory of mind score and indicates the variation in severity, as the normalized oxytocin concentration O′ varies. The severity among the individual data points in the theoretical model varies as 1/Ra′O′, where Ra′ and O′ are normalized. For fixed values of the normalized oxytocin concentration O′, the severity of individual data points varies as 1/Ra′, where Ra′ is the normalized number of available oxytocin receptors for each data point.

However, it is important to note that, for any fixed oxytocin concentration, the average value of Ru′ for the collection of data points at the fixed value of the normalized oxytocin concentration will not necessarily equal the value of Ru′ corresponding to the least square line, or autism line. This value of Ru′ is a fixed value equal to the average value of the unavailable normalized number of oxytocin receptors for all of the individual data points, which is not necessarily equal to the average values for the normalized unavailable oxytocin receptors at fixed values of the normalized oxytocin concentration O′ that are presented in the Stanford study.

The mathematical model indicated in this section above demonstrates that, for fixed values of the normalized oxytocin concentration O′, the severity of the autism Ss′ varies as the normalized number of unavailable oxytocin receptors Ru′ for that fixed normalized oxytocin concentration O′, thus establishing the significance of the oxytocin receptor in autism. The mathematical model also demonstrates that the severity of individual data points can be explained in terms of both the normalized oxytocin concentration O′ and the normalized number of available oxytocin receptors Ra′, as indicated in the theoretical model where S′ = 1/(Ra′O′) in general, and S′ goes as 1/Ra′ for fixed values of O′ as discussed above.

It is important to note the difference between the relationship Ss′ = Ru′ and the severity index (SI) = Ru′/Ra′, where Ss′ is the normalized value of the severity of autism taken from the NEPSYS theory of mind scale, and SI is a theoretical construct established for the purpose of demonstrating the equivalence between the theoretical and empirical models discussed above in establishing the significance of the oxytocin receptor in autism.

## 3. Steady-State Model for the Baseline Concentration of Oxytocin in the Cerebral Spinal Fluid and the Blood

When oxytocin binds with oxytocin receptors on the magnocellular neurons, oxytocin is produced, some of which enters the extracellular fluid surrounding the magnocellular neurons in the paraventricular and supraoptic nuclei in the hypothalamus [3–5]. For the purposes of this paper, the words extracellular fluid and cerebral spinal fluid are used interchangeably. The rate at which oxytocin binds to the oxytocin receptors of the magnocellular neurons is related to the oxytocin concentration in the vicinity of the magnocellular neurons, that is, the higher the concentration, the greater the rate at which binding of the oxytocin to the oxytocin receptors occurs. Consequently, oxytocin-oxytocin receptor binding rates may be linear relationships of oxytocin concentrations and exposure times.

This leads to the consideration that the baseline concentration of oxytocin may be a self-sustaining concentration. In other words, the rate at which oxytocin is produced from the binding of oxytocin from the baseline concentration of oxytocin to the oxytocin receptors of the magnocellular neurons and enters the extracellular fluid in the nuclei mentioned above may equal the rate at which oxytocin from the baseline oxytocin concentration is degraded together with its rate of removal via the ventricles with waste products. Oxytocin is degraded by the action of the enzyme oxytocinase, an aminopeptidase, [3]. Oxytocinase and oxytocin are found in the extracellular fluid, particularly in the paraventricular and supraoptic nuclei in the hypothalamus.

Simply put, the rate of oxytocin generation may equal the rate of oxytocin degradation together with the rate of its removal in the extracellular fluid via the ventricles with waste products. This results in a relatively constant, stable baseline concentration of oxytocin that has been observed in the cerebral spinal fluid [6]. Furthermore, the oxytocin levels in the paraventricular and supraoptic nuclei may directly influence the constant baseline oxytocin levels in the brain at large, and quite possibly in the blood. As indicated in the findings of the Bonn study [6], it was found that the baseline concentration of oxytocin in the blood and cerebral spinal fluid of the subjects was constant and averaged 6.4 pg/ml in the blood and 19.6 pg/ml in the cerebral spinal fluid of adult men throughout the course of the baseline measurements in the study. Blood samples were taken and spinal taps were done over the course of the study to provide the data on the respective oxytocin concentrations in the blood and the cerebral spinal fluid.

It should be noted that the magnocellular neurons which produce oxytocin transport it both to the blood via their axons which go to the posterior lobe of the pituitary gland and to the extracellular fluid by releasing it from their dendrites [3]. It is not known if the ratio of the oxytocin in the blood and in the extracellular fluid will remain constant for changes in the baseline concentrations of oxytocin in the extracellular fluid. However, it is noteworthy that the ratio averaged over the subjects in the Bonn study [6] remained constant, though individual concentrations of oxytocin would likely have varied. This suggests that individual ratios remained constant and that declines and/or increases in the baseline concentration of oxytocin in the extracellular fluid may be associated with declines and/or increases in the baseline concentration of oxytocin in the blood for affected individuals, which is assumed below.

In the event the numbers of oxytocin receptors on the oxytocin-producing magnocellular neurons decline, there may be less binding with the oxytocin in the cerebral spinal fluid. Less oxytocin will be produced via neuromodulation of the magnocellular neurons, and the previous baseline concentration of oxytocin will not be able to be maintained because the rate of production of oxytocin will be less than its rate of degradation by the action of oxytocinase together with its rate of removal via the ventricles with waste products. The decline in the baseline oxytocin concentration may be proportional to the decline in the number of oxytocin receptors, which implies that the normalized decline, or percentage decline, in the baseline oxytocin concentration and the number of oxytocin receptors will be the same. This may continue until the baseline concentration of oxytocin has declined to the point where the rate of oxytocin loss may equal the rate of oxytocin production.

As indicated in Section 2, the relative decrease in the baseline concentration of oxytocin may be proportional to the relative decrease in the number of available oxytocin receptors, and by inference, percent changes in the normalized values of the oxytocin concentration and in the relative number of oxytocin receptors, O′ and Ra′, remain equal. As stated above, lower baseline concentrations of oxytocin in the brain may be associated with lower baseline concentrations of oxytocin in the blood [6], and lower baseline concentrations of oxytocin in the blood are associated with increased severity of autism [1].

## 4. Oxytocin Receptor Desensitization and Downregulation before Birth

Epidemiological studies by Harvard University [7], Yale University [8], and Duke University [9] investigated the occurrence of autism among offspring, some of whose mothers received Pitocin during labor. Weak correlations between offspring autism development and Pitocin use were identified in each study, and in the study by Duke University, it was speculated that desensitization of oxytocin receptors followed by downregulation may be a cause for offspring autism development. A subsequent paper by this author [10] presented a mathematical model describing a possible process by which offspring autism development may occur in approximately 0.5% to 1% of the cases of autism, as a result of long labors with long Pitocin infusion times and high infusion rates, which could lead to desensitization and downregulation of oxytocin receptors in the fetal brain.

The mathematical model explained the weak correlations found in each of the epidemiological studies mentioned above, as arising from a desensitization threshold above which a correlation between offspring autism development and Pitocin use during labor may be found and below which no correlation exists. The mathematical model also demonstrated the significance of the half-life of oxytocin in the maternal circulation, the Pitocin infusion rate, and the maternal blood volume as reflected in the mother's weight, as factors in offspring autism development. The mathematical model developed the concept of oxytocin override of placental oxytocinase degradation of oxytocin to explain the diffusion of oxytocin from the maternal circulation across the placenta to the fetal circulation. The mathematical model is not reproduced here.

This paper called for detailed epidemiological analysis to possibly identify risk factors associated with possible offspring autism development arising from the factors indicated above, and in doing so assess the validity of the mathematical model developed in the paper. The paper called for adjustments in the use of Pitocin during labor for an at-risk population of mothers in the event the findings of a detailed epidemiological analysis validated the findings of the mathematical model. In particular, the use of pulsatile Pitocin infusion during labor was reviewed and suggested as a possible alternative to the use of continuous Pitocin infusion during labor, in the event of long labors with long infusion times with high continuous Pitocin infusion rates.

## 5. Oxytocin Receptor Formation before Birth

The presence and concentration of oxytocin receptors in the brain of the fetus at birth bears discussion. It has been found that just before birth estradiol has a very important role in influencing the number and concentration of oxytocin receptors, increasing them by as much as a factor of five [11]. Variation in the ambient concentration of estradiol may significantly influence the number and concentration of oxytocin receptors that develop in the brain of the fetus, which may be a factor in the onset of autism [12]. The expression of oxytocin receptors in the fetal brain is fixed before birth. If their development is curtailed, as shown in Section 2, then, there may be a possible reduction of the oxytocin receptors on the magnocellular neurons in the paraventricular and supraoptic nuclei in the hypothalamus, which may possibly lower the baseline oxytocin concentration. As indicated in the section above on the steady-state model, this may result in a lower concentration of oxytocin interacting with fewer oxytocin receptors, which may lead to the development of autism among the offspring.

High oxytocin concentrations in the cerebral spinal fluid may interfere with the development of new oxytocin receptors in the brain of the fetus, as they do for existing oxytocin receptors during desensitization and downregulation [7, 10]. This, in addition to the reduction in the numbers of oxytocin receptors that may result from lower levels of estradiol [11], it may influence the development and severity of autism. Similarly, the presence of progesterone in the fetal brain could inhibit the development of the oxytocin receptors, as it can do in the uterus during labor [11]. Likewise, lower levels of testosterone and/or amylase, the enzyme that converts testosterone into estradiol, may also influence the development and severity of autism because of their influence on the concentration of estradiol.

This latter concern could be more significant in males, in that females obtain most of the estradiol in their brains from their ovaries, while males obtain the estradiol in their brains from the action of the enzyme amylase on testosterone. The blood-brain barrier is permeable to estradiol and progesterone, which have molecular masses below the upper limit of 400 to 600 Daltons (Da) for transferring molecules across the blood-brain barrier [13]. Consequently, disruption of oxytocin receptor expression may occur in the fetal brain and may be caused by one or more of the factors indicated above, resulting in behavioral and neurophysiological effects that are similar to the effects associated with autism [14].

## 6. MAST Immune System Disease and Autism

MAST Immune System disease appears to result from an inflammation in the brain [15, 16]. One aspect of this inflammatory response is that the blood-brain barrier becomes more permeable [15, 16]. Another aspect of MAST Immune System disease is that it has been associated with the development of autism [15, 16]. This section will attempt to relate these two factors in terms of an earlier study of baseline oxytocin concentrations in the blood and cerebral spinal fluid [6] and a study relating the severity of autism with the variation of baseline oxytocin concentrations in the blood [1]. To do this, it is important to note that the transport of oxytocin across the blood-brain barrier may be explained by either diffusion though a porous medium or as active transport of a neuropeptide [13]. In either case, we can postulate that the movement of oxytocin across the blood-brain barrier may follow a concentration gradient from areas of higher concentration to areas of lower concentration.

From the Bonn study [6], it was found that the baseline concentration of oxytocin in the blood was 6.4 pg/ml, while the baseline concentration of oxytocin in the cerebral spinal fluid was 19.6 pg/ml. In other words, the baseline concentration of oxytocin in the cerebral spinal fluid was found to be just over a factor of 3 times as great as the baseline concentration of oxytocin in the blood. As noted above, during an occurrence of MAST Immune System disease, the blood-brain barrier becomes more permeable, permitting the movement of molecules such as oxytocin through the blood-brain barrier that would not be transported across a normal blood-brain barrier. Since the baseline concentration of oxytocin is higher in the cerebral spinal fluid than in the blood, it is likely that oxytocin will follow the reverse concentration gradient and move from the cerebral spinal fluid into the blood until the concentrations are equilibrated. Since the volume of the blood is much higher than the volume of cerebral spinal fluid in the brain, the baseline concentration in the blood will have a modest increase.

However, calculations indicate that the baseline concentration of oxytocin in the cerebral spinal fluid will decline by approximately 60%, which may place the affected individuals in the autistic range. For the purposes of assessing the severity of autism, a decline in the baseline oxytocin concentration in the cerebral spinal fluid of the affected individuals of 60% may correspond in theory with a similar decline in the baseline concentration of oxytocin in the blood. This correspondence may be suggested by the constant ratio between the baseline oxytocin concentrations in the blood and in the cerebral spinal fluid indicated in the Bonn study [6], as discussed in the section above on the steady-state model. The NEPSYS theory of mind score, a test for autism, has been correlated to baseline oxytocin blood levels in children [1]. If this constant ratio in baseline oxytocin concentrations in the blood and cerebral spinal fluid is the case, a 60% decline in the oxytocin concentration in the cerebral spinal fluid would result in a significantly lower NEPSYS theory of mind score for the affected individuals, possibly as much as 60%, placing them in the autistic range.

Some calculations may be helpful. An oxytocin concentration of 6.4 pg/ml in 4.7*E*3 ml of blood (the volume of blood in an average woman [10]) = 3.008*E*4 pg. An oxytocin concentration of 19.6 pg/ml in 4*E*2 ml of cerebral spinal fluid (the approximate volume of cerebral spinal fluid in an adult brain, in which the volume of the cerebral spinal fluid includes the volume of the extracellular fluid) = 7.84*E*3 pg. So, the average concentration in the blood and cerebral spinal fluid equilibrates and becomes (3.008*E*4 + 7.84*E*3) pg/(4.7*E*3 + 4*E*2) ml = 3.792*E*4 pg/5.1*E*3 ml = 7.4 pg/ml. The concentration of oxytocin in the cerebral spinal fluid declines by 62% from 19.6 pg/ml to 7.4 pg/ml, while the concentration of oxytocin in the blood increases by 16% from 6.4 pg/ml to 7.4 pg/ml. From the discussion in the section above on the steady-state model, the reduction of the concentration of oxytocin by 62% would result in a new lower baseline concentration of oxytocin for as long as the increased permeability of the blood-brain barrier resulting from the MAST Immune System disease existed. This decrease in oxytocin concentration in the cerebral spinal fluid may be more pronounced in men, who for the purposes of the calculation in this section have a larger average blood volume that more than compensates for the larger average volume of their brains.

The relationship of this reduction in the baseline concentration of oxytocin in the cerebral spinal fluid to the development of autism may be associated with the findings of the Stanford study [1], which demonstrated the linear relationship between oxytocin concentration in the blood and scores on the NEPSYS theory of mind scale. Lower concentrations of oxytocin in the blood correlated with lower scores on the NEPSYS theory of mind scale, with lower scores indicating greater severity of autism. From the considerations discussed above in Section 3, a reduction of the baseline concentration of oxytocin in the cerebral spinal fluid of approximately 60% may be associated with a similar reduction in the baseline concentration of oxytocin in the blood, and hence, it may be associated with a similar decline in the NEPSYS theory of mind score. This reduction in the baseline concentration of oxytocin may lead to the onset of autism, or what may appear as symptoms of autism. Consequently, individuals with MAST Immune System disease may have NEPSYS theory of mind scores that have declined enough to indicate that these individuals are in the autistic range.

## 7. Recent Findings Relating an Excess of Dendritic Spines with Autism

Recent research has demonstrated that an excess of dendritic spines are associated with autism that results from an absence of pruning of the dendritic spines [17]. It has also been found that the dendritic spines of autistic individuals are damaged [17, 18]. The connection to autism may lie in part in the possible interruption of neuromodulation by oxytocin of oxytocin receptors on the dendrites, in that damaged dendritic spines have been associated with cellular residues in the neurons [17, 18]. The presence of cellular residues may suggest dysfunction in the cellular function of the neurons, in particular, with their signaling, which may diminish their capability for neuromodulation. It is beyond the scope of this paper to address this possible dysfunction of the dendritic spines of autistic individuals at greater length. This awaits further research.

However, the interruption or limitation of the neuromodulation by oxytocin of oxytocin receptors in the dendrites resulting from damaged dendritic spines, if it occurs, may be interpreted in a similar way as a reduction in the number of available oxytocin receptors in fully functional neurons, which would also limit or disrupt the neuromodulation by oxytocin. This would indicate a disruption in the functioning of the oxytocin-oxytocin receptor system. As discussed earlier in this paper in Section 2, a reduction in the number of available oxytocin receptors may be a significant factor in the development and severity of autism, which may also be true for a disruption of the neuromodulation of oxytocin receptors by oxytocin in damaged neurons. If the magnocellular neurons are damaged, lower baseline concentrations of oxytocin may result, which may magnify the severity of the resulting autism, in that a lower concentration of oxytocin will be interacting with oxytocin receptors on damaged neurons, in which neuromodulation by oxytocin of the oxytocin receptor system may be compromised.

It is noteworthy that much current thought about autism focuses on neural circuits and synapses as factors in autism [19]. Indeed, autism is thought of as the disease of the synapse. Autism is thought to encompass a wide range of neurological disorders with developmental origins and all of them including the characteristic of impaired social interaction and repetitive behaviors [19]. This raises an intriguing question of how distinct alterations of neural circuits during different developmental stages lead to common behavioral manifestations in autism, including lack of social communication and repetitive behaviors. A recent study by Stanford University [20] demonstrated that intranasal oxytocin augmentation significantly enhanced social abilities in children with autism. Children with the lowest concentrations of oxytocin in their blood showed the greatest social improvement. Oxytocin's effects were specific to social functioning with no decrease in repetitive behaviors [20]. An earlier study by Stanford University related severity of autism to decreases in oxytocin concentrations in the blood of children [1].

From the above considerations, the mathematical models and explanations presented in this paper demonstrate that decreases of oxytocin and oxytocin receptors may provide an explanation for the ubiquitous presence of impaired social interaction in all the neurological disorders encompassing autism, whereas the ubiquitous presence of repetitive behavior would have other explanations, perhaps related to neural circuitry and/or synaptic dysfunction. The pruning of excess dendritic spines may, in theory, improve the signaling capability of the affected neurons. If this occurs, it may result in enhanced neuromodulation in these neurons, and as discussed in Section 3, in the case of magnocellular neurons, an increase in oxytocin production and oxytocin concentration in the cerebral spinal fluid with consequent improvements was observed in social functioning.

## 8. Conclusions

Mathematical operations on normalized data from the Stanford study, relating the normalized severity of autism with the normalized concentration of oxytocin in children, are shown to be equivalent to a theoretical expression of the severity of autism. The severity of the autism in the theoretical model is inversely proportional to the normalized number of oxytocin receptors for a fixed normalized oxytocin concentration, in which the severity of the autism is zeroed to the horizontal or *X*-axis. This equivalence establishes the validity of the theoretical model and interprets the Stanford data as indicating the significance of the oxytocin receptor in the severity of autism as measured by the NEPSYS theory of mind scores used in the Stanford study.

A steady-state model is presented to explain the constant baseline concentration of oxytocin in the cerebral spinal fluid and blood observed in the subjects of the Bonn study. The model is based on the consideration that oxytocin from the baseline concentration of oxytocin in the cerebral spinal fluid binds with oxytocin receptors on the magnocellular neurons in the hypothalamus and may produce the same quantity of oxytocin that is degraded by the action of oxytocinase as well as removed with waste products via the ventricles. This constant baseline concentration of oxytocin in the cerebral spinal fluid may be associated with the constant baseline concentration of oxytocin in the blood. Decreases in the baseline concentration of oxytocin in the cerebral spinal fluid, and by extension in the blood, are explained in terms of a proportional decrease in the number of oxytocin receptors in the magnocellular neurons in nuclei in the hypothalamus.

Offspring autism development, as a consequence of high levels of Pitocin infusion during long labors with long Pitocin infusion times, may result in the desensitization and downregulation of oxytocin receptors in the fetal brain, is reviewed in light of recent epidemiological studies by Harvard, Yale, and Duke universities, with reference to an earlier paper by the author presenting a mathematical model as to how this might occur. The mathematical model demonstrates the significance of oxytocin half-life in the maternal circulation as well as Pitocin infusion rate and mother's weight as factors in offspring autism development. It introduces the concept of a desensitization threshold to explain weak correlations of Pitocin use during labor with offspring autism development identified in each of the epidemiological studies mentioned above, which may account for 0.5% to 1.0% of the cases of autism arising each year. The paper calls for a detailed epidemiological analysis to assess the validity of the mathematical model and, if valid, identify risk factors for offspring autism development. Pulsatile Pitocin infusion is reviewed as a possible alternative to continuous Pitocin infusion to mitigate the possible risk of offspring autism development arising from labors with high Pitocin infusion rates over long Pitocin infusion times.

The impact of a reduction of the number of oxytocin receptors comprising the fixed expression of oxytocin receptors before birth is explained as a possible factor in the onset of autism development in the offspring. Several methods by which this could occur are examined in terms of their negative impact on the numbers and formation of oxytocin receptors. Particular attention is given to the possible reduction of the oxytocin receptors on the magnocellular neurons in nuclei in the hypothalamus, leading to a reduction in the concentration of oxytocin in the cerebral spinal fluid, which is explained in Section 3 for baseline oxytocin concentration given above. As indicated in the steady-state model, a reduction of the oxytocin concentration in the cerebral spinal fluid, as well as declines in the number of oxytocin receptors, in which neuromodulation may occur, may lead to the possibility of offspring autism development.

The association of autism with MAST Immune System disease is explained in terms of a reverse transfer of oxytocin through the blood-brain barrier, which becomes more permeable as a result of the MAST Immune System disease, from a higher concentration of oxytocin in the cerebral spinal fluid to a lower concentration of oxytocin in the blood. The resulting concentration of oxytocin in the cerebral spinal fluid is shown to undergo a possible decrease of approximately 60% in women of average size, which may be shown to place them in the autistic range, by comparison with a 60% decline in oxytocin concentration in the blood and the corresponding decline in the NEPSYS theory of mind scores. There may be a more pronounced decrease of oxytocin concentration in the cerebral spinal fluid of men of average size.

Recent findings relating an excess of dendritic spines, which have been damaged and failed to undergo pruning, with the presence of autism is explained partially in terms of the possible negative impact on the neuromodulation of oxytocin receptors. If the dendrites of the magnocellular neurons in the hypothalamus are damaged and the neuromodulation of oxytocin receptors is negatively impacted, then a subsequent decline in the baseline concentration of oxytocin may occur. This may magnify the severity of the resulting autism resulting from the disruption of the neuromodulation in other neurons, that is, in other words, it is a situation where a lower baseline concentration of oxytocin interacts with oxytocin receptors on damaged neurons, in which the neuromodulation of the oxytocin receptors may be negatively impacted. Pruning of the dendritic spines may improve signaling in the affected neurons, which may result in enhanced neuromodulation and, in the case of magnocellular neurons, increases in oxytocin production and oxytocin concentration in the cerebral spinal fluid with consequent improvements in social functioning.

This paper asserts that oxytocin and oxytocin receptors may be a common denominator associated with the causes of autism. The possible significance of oxytocin receptors, which is indicated in this paper in the development of autism attributed to the diseases and causes mentioned above, should be motivation for further research into medical treatments that may possibly restore the number and functioning of oxytocin receptors that may be impacted by these diseases and consequently possibly alleviate the severity of the autism that is associated with these diseases. One plausible avenue of research may be the possible generation of magnocellular neurons and other neurons from adult stem cells of autistic individuals for *in vitro* study of the oxytocin receptor and its function in neuromodulation. This may also serve as a basis to assess the validity of the theories presented in this paper. Other avenues of research might include research into restoring the integrity of the blood-brain barrier in MAST Immune System disease and possible restoration of neuromodulation in the neurons that may be damaged from an absence of pruning that is associated with autism. Intranasal applications of oxytocin may also be useful in alleviating the symptoms of autism identified in some of the diseases mentioned above.

While this paper addressed the variation in oxytocin concentration and relative numbers of oxytocin receptors as factors indicated in the social deficit expression of autism, it should be noted that variation in the oxytocin concentration in the cerebral spinal fluid may also impact oxytocin's role as a neurotransmitter in the synapses. This may also be a factor in autism. However, it is beyond the scope of this paper to address this consideration.

## Figures and Tables

**Table 1:** Calculation of data points for the theoretical model in which SI = Ru′/Ra′.

Ra′	Ru′	SI = Ru′/Ra′ = (1/Ra′) − 1
0.1	0.9	9
0.2	0.8	4
0.3	0.7	2.33
0.4	0.6	1.5
0.5	0.5	1
0.6	0.4	0.67
0.7	0.3	0.43
0.8	0.2	0.25
0.9	0.1	0.11
1.0	0.0	0.0

**Table 2:** Calculation of data points from Stanford data where SI = Ss′/O′.

O	O′ = O/9	Ss (NEPSYS)	Ss′ = Ss/3.6	SI = Ss′/O′
1	0.11	3.2	0.89	8.09
2	0.22	2.8	0.78	3.55
3	0.33	2.4	0.67	2.03
4	0.44	2.0	0.56	1.27
5	0.56	1.6	0.44	0.79
6	0.67	1.2	0.33	0.49
7	0.78	0.8	0.22	0.28
8	0.89	0.4	0.11	0.12
9	1.00	0.0	0.00	0.00

## Data Availability

The underlying data in this manuscript were taken from the study by Stanford University and are cited in this study.
